# Constitutive Activation of IKK2/NF-κB Impairs Osteogenesis and Skeletal Development

**DOI:** 10.1371/journal.pone.0091421

**Published:** 2014-03-11

**Authors:** Gaurav Swarnkar, Kaihua Zhang, Gabriel Mbalaviele, Fanxin Long, Yousef Abu-Amer

**Affiliations:** 1 Department of Orthopedics, Washington University School of Medicine, Saint Louis, Missouri, United States of America; 2 Department of Medicine, Washington University School of Medicine, Saint Louis, Missouri, United States of America; University of Rochester, United States of America

## Abstract

Pathologic conditions impair bone homeostasis. The transcription factor NF-κB regulates bone homeostasis and is central to bone pathologies. Whereas contribution of NF-κB to heightened osteoclast activity is well-documented, the mechanisms underlying NF-κB impact on chondrocytes and osteoblasts are scarce. In this study, we examined the effect of constitutively active IKK2 (IKK2ca) on chondrogenic and osteogenic differentiation. We show that retroviral IKK2ca but not GFP, IKK2WT, or the inactive IKK2 forms IKK2KM and IKK2SSAA, strongly suppressed osteogenesis and chondrogenesis, in vitro. In order to explore the effect of constitutive NF-κB activation on bone formation in vivo, we activated this pathway in a conditional fashion. Specifically, we crossed the R26StopIKK2ca mice with mice carrying the Col2-cre in order to express IKK2ca in osteoblasts and chondrocytes. Both chondrocytes and osteoblasts derived from Col2Cre/IKK2ca expressed IKK2ca. Mice were born alive yet died shortly thereafter. Histologically, newborn Col2Cre+/RosaIKK2ca heterozygotes (Cre+IKK2ca_w/f (het)) and homozygotes (Cre+IKK2ca_f/f (KI)) showed smaller skeleton, deformed vertebrate and reduced or missing digit ossification. The width of neural arches, as well as ossification in vertebral bodies of Cre+IKK2ca_w/f and Cre+IKK2ca_f/f, was reduced or diminished. H&E staining of proximal tibia from new born pups revealed that Cre+IKK2ca_f/f displayed disorganized hypertrophic zones within the smaller epiphysis. Micro-CT analysis indicated that 4-wk old Cre+IKK2ca_w/f has abnormal trabecular bone in proximal tibia compared to WT littermates. Mechanistically, ex-vivo experiments showed that expression of differentiation markers in calvarial osteoblasts derived from newborn IKK2ca knock-in mice was diminished compared to WT-derived cells. In situ hybridization studies demonstrated that the hypertrophic chondrocyte marker type-X collagen, the pre-hypertrophic chondrocyte markers Indian hedgehog and alkaline phosphatase, and the early markers Aggrecan and type-II collagen were reduced in Cre+IKK2ca_w/f and Cre+IKK2ca_f/f mice. Altogether, the in-vitro, in vivo and ex-vivo evidence suggest that IKK2ca perturbs osteoblast and chondrocyte maturation and impairs skeletal development.

## Introduction

Bone is constantly remodeled temporally and spatially by precise regulatory mechanisms that coordinate bone formation and bone resorption [1]–[3]. Accrual of bone mass is determined by net balance between bone formation and bone resorption. Conversely, imbalance between bone resorption and bone formation leads to skeletal deformities such as bone loss (all forms of osteoporosis, osteopenia, etc) or excessive bone formation often non-remodelled as evident in various forms of osteopetrosis [4]. At the cellular level, mesenchyme-derived osteoblasts lay down matrix and hematopoietic-derived osteoclast resorb and remodel the formed bone tissue. Numerous paracrine and autocrine factors and systems regulate this process [3].

The effect of inflammatory responses on bone health has been widely described [5]–[11] and in fact, osteoporosis has been considered as a co-morbidity in patients suffering from chronic inflammatory diseases such as rheumatoid arthritis, inflammatory bowel disease (IBD), colitis, etc. [5]–[11], which typically present increased fracture risk. At the cellular level, inflammatory mediators target the entire milieu of the bone tissue; they promote differentiation of myeloid cells into osteoclasts to exacerbate bone resorption and negatively impact bone formation by targeting mesenchymal and osteoblast cells. The former effect on osteoclasts has been widely detailed [6], [12]. However, the mechanism underpinning inhibition of bone formation remains vague. In this regard, numerous clinical case reports correlated high circulating levels of inflammatory cytokines including TNF, IL-1β, IL-17, IL-4, IL-6 and others, with the bone phenotype of the subjects [13]–[15]. In other studies, elevated levels of the WNT pathway antagonists sclerostin and DKK1 were reported in animal models of rheumatoid arthritis [16]. Expression of sclerostin and DKK1 was elevated in synovial tissue from rheumatoid arthritis patients compared to controls and bone repair was often delayed or repressed in patients with systemic inflammatory background [17]–[19].

The transcription factor NF-κB has been implicated as crucial mediator of immune/inflammatory responses and required for skeletal development [20]–[25]. In this regard, it has been shown that NF-κB signaling regulates osteoclastogenesis and mediates inflammatory bone diseases [26]. IKK2, also known as IKKβ, is required for activation of the classical NF-κB pathway and mediates the vast majority of inflammatory responses [24], [27]–[30]. Constitutively active IKK2 (in which the activation loop serines are substituted with glutamic acid) sustains heightened NF-κB activity and intrinsically recapitulates the inflammatory response [31]–[33]. In this regard, we have shown that knock-in of this constitutively active form of IKK2 in the myeloid lineage in mice induced systemic osteolysis owing to elevated endogenous osteoclastogenesis [31]. However, given the ubiquitous expression of IKK2 in all tissues of mammals, the effect of IKK2 on other crucial skeletal components/processes such as osteogenesis and chondrogenesis remains elusive. Few studies suggest that cross-talk between NF-κB signaling and osteogenesis indeed exists. In fact, a recent study utilizing dominant negative approach suggests that IKK2 is a repressor of osteogenesis [34]. Another recent report has shown that NF-κB inhibits osteogenesis by promoting degradation of β-catenin, a downstream mediator of WNT signaling [35]. In other systems, it has been suggested that NF-κB activates Notch and blocks WNT signaling pathways [36]–[38]. Despite these studies, the mechanisms underlying NF-κB regulation of osteogenesis remain scant. To gain better appreciation of the developmental role of IKK2 in skeletal homeostasis and its osteo-inflammatory role, we examined the effect of constitutively active IKK2 (resembling sustained inflammatory response) during mesenchymal and chondrogenic development.

## Methods

### Mice

Coll2-cre and pROSA-IKK2ca mice have been described [33], [39]. Briefly, the pRosa26-IKK2ca transgenic mice harbor a cDNA encoding IKK2 containing two serine to glutamate substitutions in the activation loop of the kinase domain (SS→EE), preceded by a loxP-flanked STOP cassette, into the ubiquitously expressed ROSA26 locus. We crossed mice carrying this allele to the Col2-cre mice in order to express IKK2ca in osteoblasts and chondrocytes. For in vitro studies, a retrovirus expressing IKK2ca was used to transduce primary osteoblasts and transformed OB/stromal cells.

### Skeletal Analysis and Histology

For skeletal staining, newborn pups were isolated, de-skinned, eviscerated, and fixed in 95% ethanol overnight. The skeletons were then stained for 3 days with 0.3% Alcian Blue and 0.3% Alizarin Red dissolved in 75% ethanol–20% glacial acetic acid. Thereafter, the skeletons were rinsed with 95% Ethanol for 2 hrs. Samples were cleared in 1% KOH for 4 days. The skeletons were subsequently transferred to decreasing concentrations of KOH (1.6, 1.2, 0.8, and 0.4%) mixed with increasing amounts of glycerol (20, 40, 60, and 80%) at 1-day intervals. For histology, limbs were fixed in formalin, decalcified, and embedded in paraffin. Sections were cut every 6 um and stained with hematoxylin and eosin.

### In situ Hybridization

Embryonic tissues were fixed in 10% formalin overnight at room temperature, then decalcified and embedded in paraffin prior to sectioning at 6 µm. In situ hybridization was performed by using digoxigenin-labeled riboprobes as previously described [40]. Type-I a1, type-II and type-X collagen cRNA anti-sense probes were derived by in vitro transcription.

### Retroviral Infection

Retrovirus was generated by transfection of pMX-IKK2ca into Plat-E packaging cells with Fugene 6. After retroviral infection for 12–24 hours and puromycin selection for 2 days, ST2 cells or ATDC5 cells were cultured in regular complete medium for an additional 1 day before harvesting for differentiation assay.

### Primary cOB Culture

Calvaria were harvested from neonatal mice pups. Briefly, calvaria were surgically removed from the skull, sutures were removed, and adherent tissue material was cleaned by gentle scrapping using a scalpel, followed by chopping to very small fragments. Pooled calvaria were subjected to sequential digestions (15 minutes/digestion) with 0.1% dispase and 0.1% collagenase P to release cells. After discarding the cells from the first digestion (heterogeneous population), cells from the next four digestions were pooled and cultured in α ascorbic acid free-modified essential medium (α-MEM) containing 10% FBS [41].

### ST2 Cell Culture, AP Staining and von Kossa Staining

ST-2 mouse bone marrow stromal cell line (Riken Cell Bank, Tsukuba, Japan) were cultured in Dulbecco’s modified Eagle’s medium (DMEM) supplemented with 10% heat-inactivated fetal bovine serum (Invitrogen) and antibiotics. ST2 were cultured with Wnt3a conditioned medium for 2–5 days for osteoblast differentiation. For alkaline phosphatase and von Kossa stainings, cells were fixed with 10% formalin for 10 min and washed three times with 10 mM Tris⋅HCl, pH 7.2. Fixed cells were subjected to staining for alkaline phosphatase and von Kossa staining. Alkaline phosphatase was stained with naphthol AS-MX phosphate and fast blue BB salt (Sigma kit# 85L-2). The von Kossa staining for calcium was performed as follows. Fixed cells were incubated with 5% silver nitrate for 5 min in daylight, washed twice with H2O, and then treated with 5% sodium thiosulfate. Mineralized nodules were counted under a microscope.

### ATDC5 Cell Culture and Alcian Blue Staining

ATDC5 cells [42] (Riken Cell Bank, Tsukuba, Japan), derived from mouse embryonal carcinoma, were cultured in a 1∶1 mixture of Dulbecco’s modified Eagle’s medium and Ham’s F-12 (Cambrex Bio Science Inc, Walkersville, MD) supplemented with 5% fetal bovine serum (Invitrogen), 10 ug/ml human transferrin (Sigma-Aldrich), 3 X10–8 M sodium selenite (Sigma-Aldrich) at 37°C under 5% CO2. Upon reaching confluence, the cells were stimulated with 10 ug/ml of bovine insulin (Sigma-Aldrich), and the medium was changed every other day. Alcian blue (Sigma-Aldrich), which stains cartilage-like extracellular matrix produced by mature chondrocytes, was used to evaluate expression of extracellular matrix by ATDC5 cells. The staining was done after the cells were rinsed with phosphate-buffered saline, incubated with 0.1% Alcian blue solution in 0.1 N HCl (pH 1.0) overnight, and washed three times with phosphate-buffered saline before macroscopic examination.

### Reverse Transcription-polymerase Chain Reaction Analysis of Osteoblast, Chondrocyte Differentiation

ST2 cells and ATDC5 cells treated for Wnt3a or insulin for the indicated periods were subjected to RT-PCR with the specific primers. Osteocalcin (OSC) and aggrecan are osteoblast-specific secretory protein and extracellular matrix protein in cartilage respectively. GAPDH was used as internal control.

This study was carried out in strict accordance with the recommendations in the guide for the Care and Use of Laboratory Animals of the National Institute of Health. All animals are housed in the facility at Washington University under the care of trained technicians and veterinarians. Animals experiencing pain or discomfort following procedures are treated with analgesics per approved protocol. Euthanasia is performed by inhalation of gaseous CO2 in a closed chamber. This method is approved by the AVMA. All animal work was pre-approved by the animal committee of Washington University and conducted according to AVMA guidelines.

### Statistical Analysis

Data are expressed as mean ± SEM unless otherwise indicated. The data obtained in experiments with multiple treatments were analyzed using one-way ANOVA followed by post hoc Newman-Keuls test of significance or student t-test. Qualitative observations have been represented following triple blind assessments. All experiments were repeated at least three times and quantitative data for in vivo data were collected from groups of at least 6 mice per group.

## Results

### Constitutive Activation of NF-κB in Osteoblasts, Chondrocytes and Stromal Cells Inhibits their Differentiation

**Figure 1:**
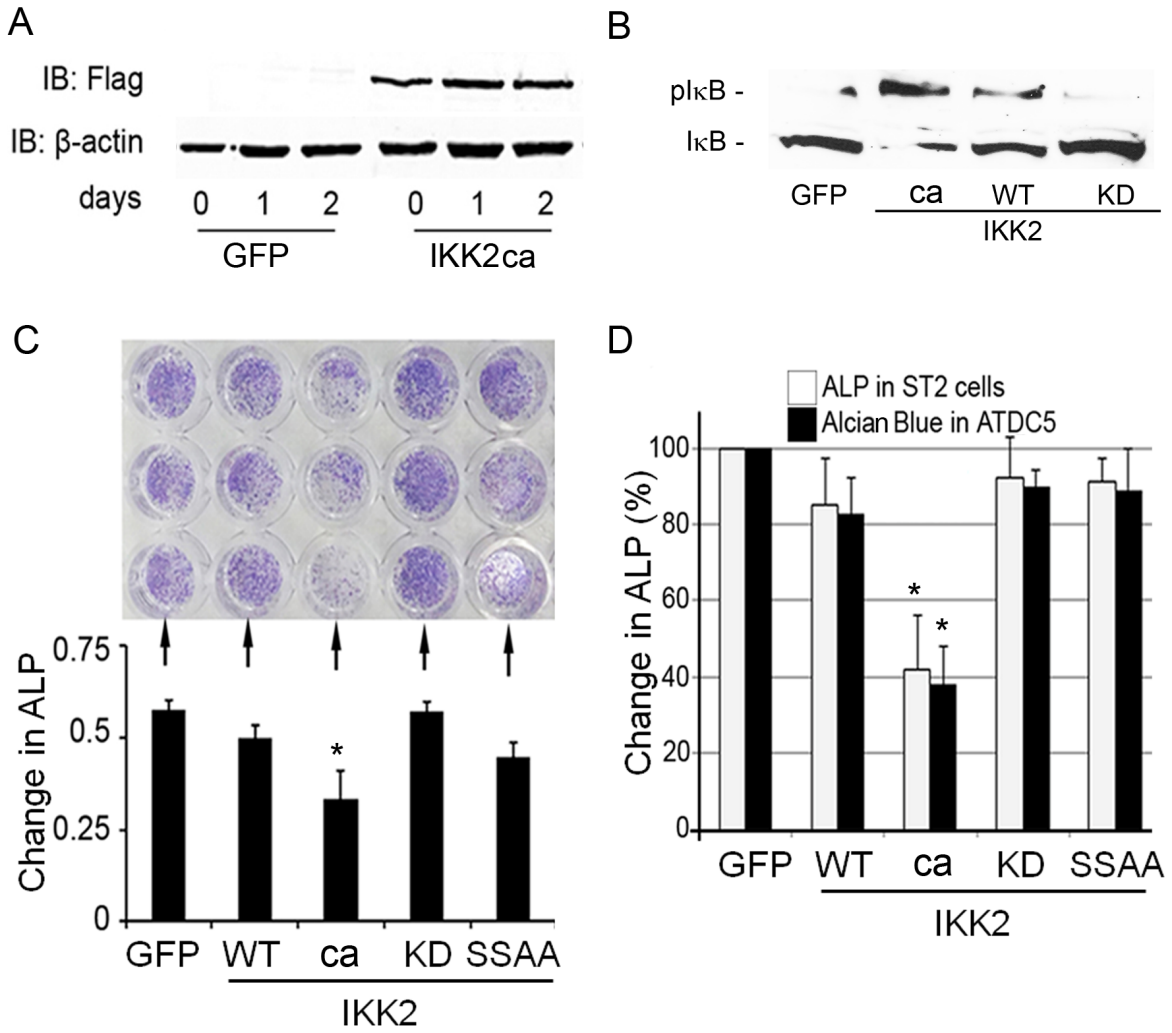
Constitutive activation of NF-κB in osteoblasts, chondrocytes and stromal cells inhibits their differentiation. (A) Calvarial osteoblasts (cOB) were transduced using retroviral constructs pMX-GFP and pMX-IKK2-ca and incubated for the time points indicated. Western blots were used to show expression of Flag-tagged IKK2 and β-actin in cOB. (B) Expression of IκB and phospho-IκB in lysates of OBs transduced with pMx-GFP or the various forms of IKK2 as indicated. (C–D) Calvarial OBs (C), ST2 and ATDC5 cells (D) were infected with GFP, IKK2WT, IKK2ca, IKK2KD, or IKK2SSAA and incubated for 21 days with 10 ug/ml of bovine insulin (media was changed and supplemented with fresh insulin every 48 hrs). Cells were then stained with either alkaline phosphatase (ALP) or Alcian blue. Note reduced staining in IKK2ca conditions. Lower left panel (C) depicts optical density quantification of upper panel. Right panel (D) represents quantification of ST2 cell and ATDC5 staining (not shown) using arbitrary units expressed as % of control. Experiments were repeated at least three times in triplicate conditions. Asterisk represents p<0.01.

Osteoblasts and chondrocytes are derived from mesenchymal precursor cells and are the principal bone and cartilage forming cells, respectively. Persistent NF-κB activation is central to inflammatory responses that impact all components of bone development. To mimic the impact of inflammatory responses on osteoblasts, we virally expressed IKK2ca (FLAG-tagged) in calvarial osteoblasts and demonstrated its expression and activity (fig. 1A–B) as evident by significant phosphorylation of the substrate IκB by IKK2ca compared to minimal or absence of IκB phosphorylation (pIκB) by wild type (WT) or kinase-dead (KD) IKK2, respectively (1B). Note that IκB phosphorylation coincides with its degradation (Fig. 1B; lane 2).

To examine the effect of NF-κB activation on their differentiation and maturation, the bone marrow stromal cell line ST2 cells and the chondrogenic cell line ATDC5 were infected with viral particles expressing constitutively active IKK2 (referred to as “ca”), control GFP, wild type IKK2 (WT), kinase dead (KD) IKK2, and phospho-deficient (SSAA) IKK2. The results indicate that expression of the stromal cell differentiation marker alkaline phosphatase and the chondrocyte differentiation marker Alcian blue are significantly blunted by IKK2ca (ca) (fig. 1C–D). In contrast, no change was observed with GFP, WT, or SSAA transduction. Consistently, mRNA expression of alkaline phosphatase in ST2 cells and chondrocyte markers (Sox9, Col2A1, aggrecan) in ATDC were reduced in IKK2ca-infected cells compared with GFP or IKK2WT-infected cells (Fig. S1).

### Mice Expressing IKK2ca Exhibit Abnormal Skeletal Development

**Figure 2:**
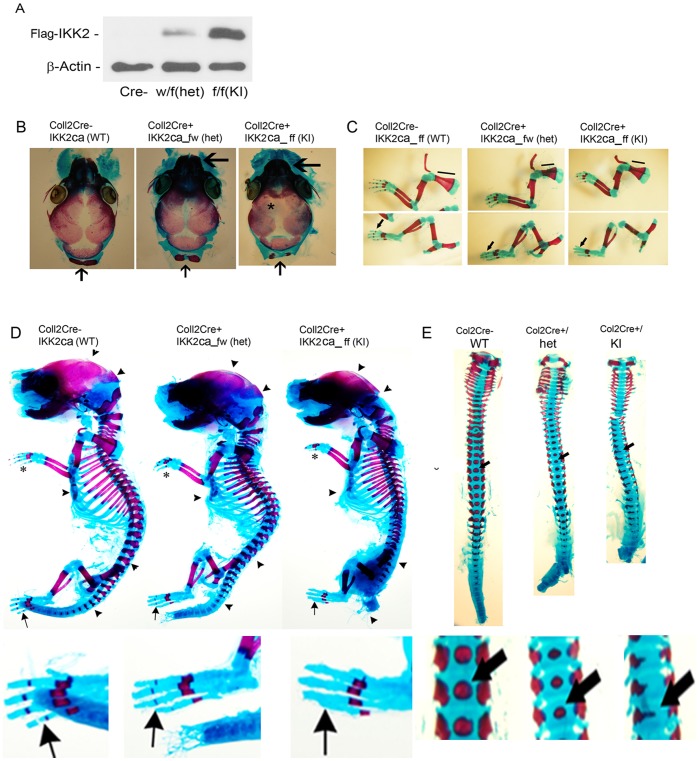
Mice expressing IKK2ca exhibit abnormal skeletal development. (A) cOB cells were harvested form newborn mice, lysed and subjected to Western blot with Flag (IKK2) and beta-actin antibodies. (B) Neurocranium of Newborn WT, Col2Cre+/IKK2ca heterozygotes (het) and homozygotes (KI). Sutures and fontanelles are widened in Col2Cre+/IKK2ca homozygotes (asterisk). Arrows (bottom) indicate the unfused and smaller supraoccipital bone in Col2Cre+/IKK2ca heterozygotes and homozygotes, respectively. Shortened snout in homozygous knock-in (large arrow) compared with hets and WT littermates. (C–D) Newborn pups were stained with Alcian blue/Alizarin red. Bone is stained red and cartilage blue. Limbs showed reduced length of scapula in newborn Col2Cre+/IKK2ca heterozygotes and homozygotes (D) Smaller skeleton and deformed vertebrate in newborn Col2Cre+/IKK2ca heterozygotes (wf) and homozygotes (ff). Reduced or missing digit ossification in Col2Cre+/IKK2ca heterozygotes and homozygotes respectively (arrows and asterisks). Reduced or diminished ossification in vertebral bodies and skull in knock-in mice is apparent (arrow heads). (E) Dorsoventral view of vertebra showing reduced width of neural arches as well as reduced or diminished degrees of ossification in vertebral bodies (arrow) of newborn Col2Cre+/IKK2ca heterozygotes and homozygotes respectively compared to WT littermates.

In order to explore the role of constitutive NF-κB activation during bone formation in vivo, we decided to activate this pathway in a conditional fashion. We employed the R26StopIKK2ca mice in which a cDNA encoding IKK2 containing two serine to glutamate substitutions in the activation loop of the kinase domain, preceded by a loxP-flanked STOP cassette, was cloned into the ubiquitously expressed ROSA26 locus [33]. We crossed mice carrying this allele to the Col2-cre mice in order to express IKK2ca in chondrocytes, as well as osteoblasts. Both chondrocytes (not shown) and osteoblasts (fig. 2A) derived from Cre+IKK2ca_w/f or Cre+IKK2ca_f/f express Flag-tagged IKK2ca detected by Western blot. Histologically, newborn Cre+IKK2ca_w/f heterozygotes Cre+IKK2ca_f/f and homozygotes showed smaller skeleton and deformed vertebrates. Examination of the appendicular and axial skeleton revealed reduced or missing digit ossification (arrows and asterisks) in heterozygotes and homozygotes, respectively (fig. 2C–D). Compared to WT littermates, the width of neural arches as well as ossification in vertebral bodies of IKK2ca heterozygotes and homozygotes were reduced or diminished (fig. 2E; arrows). Sternum with ribs showed wider xiphoid process with two well-separated ossification centers in newborn IKK2ca heterozygotes and homozygotes (not shown). Reduced length of scapula in newborn IKK2ca heterozygotes (26±5%) and homozygotes (39±4%) was also noted (fig. 2C; horizontal lines). In addition, sutures and fontanelles were widened in IKK2ca homozygotes (fig. 2B; arrows, 2D; arrow heads). IKK2ca homozygote mice died shortly after birth, while heterozygote mice survived to maturity.

### IKK2ca Expression in vivo Dampens Expression of Chondrogenic and Osteogenic Markers

**Figure 3:**
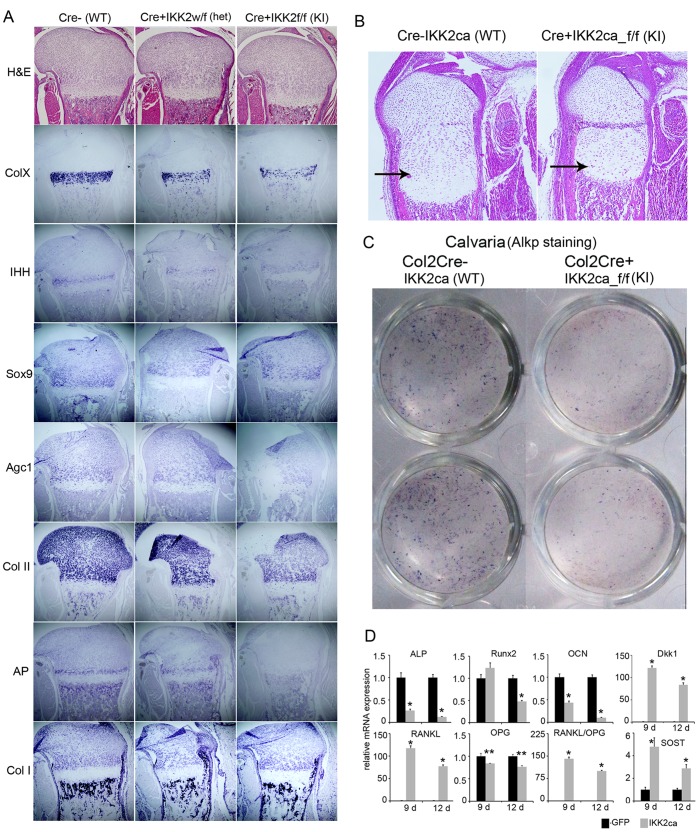
IKK2ca expression in vivo dampens expression of chondrogenic and osteogenic markers. (A) Hematoxylin and eosin (H&E) staining of proximal tibia from new born pups. Col2Cre+/IKK2ca heterozygotes and homozygotes displayed disorganization of hypertrophic zones within the smaller epiphysis compared to WT littermates. In situ detection of type-I a1(Col I) type-II (Col II), type-X collagen (Col X), Alkaline phosphatase (AP), indian hedgehog (Ihh), Sox 9, and Aggrecan (Agc1) mRNAs in growth plates of femur sections prepared from new born Col2Cre+/IKK2ca homozygotes, heterozygote and control littermates. Reduced Col I, Col-II, AP positive osteoblast and Col X, Sox9 hypertrophic chondrocytes in Col2Cre+/IKK2ca heterozygote, compared to WT control is noted. (B) Higher magnification of H&E-stained sections for new born wild type and Cre+IKK2ca homozygotes. Columnar zones are indicated with arrows (C) Alkaline phosphatase staining of cOB isolated from WT and IKK2ca knock-in mice. (D) IKK2ca affects the mRNA expression of various osteoblast-differentiation associated genes. cOB were cultured in differentiation media for 9 and 12 days, followed by mRNA isolation and qPCR for various osteoblast differentiation associated gene. While, IKK2ca expression decreases the mRNA expression of ALP, Runx2 and OCN (asterisk indicates p<0.01), it increases the mRNA expression of DKK1 and SOST which negatively regulates Wnt signaling. It also increases the RANKL/OPG ratio by increasing the expression of RANKL. Asterisks * and ** represent p<0.01 and 0.05, respectively. All experiments including the histology and qPCR were repeated 3 times (n = 3).

In order to further characterize the molecular mechanism underlying the effect of constitutively active IKK2 in the regulation of osteogenesis and chondrogenesis, we performed in situ hybridization of several markers for either chondrogenesis or osteogenesis in new born transgenic mice and control littermates. Hematoxylin and eosin (H&E) staining of proximal tibia from new born pups showed that Cre+/RosaIKK2ca homozygotes displayed disorganized hypertrophic zones within the smaller epiphysis (columnar zone) compared to WT littermates (fig. 3A,B; arrows). We found that the hypertrophic chondrocyte marker type-X collagen (Col X) was reduced or diminished in IKK2ca heterozygote or homozygote, compared to WT control (fig. 3A). The pre-hypertrophic chondrocyte markers Indian hedgehog (Ihh) and alkaline phosphatase were also significantly reduced or lost in IKK2ca heterozygote or homozygotes. The early differentiation markers such as Aggrecan and type-II collagen (Col II) were reduced, while Sox9 only slightly reduced in IKK2ca homozygote mice, compared to WT controls (fig. 3A). In all these circumstance, HE staining showed that there is no obvious cell loss in cartilage, suggesting that IKK2 suppresses chondrocyte differentiation. Although Type-I a1(Col I) mRNA remained unchanged, AP mRNA signal was diminished in osteoblast derived from IKK2ca homozygote compared to WT controls (fig. 3A).

Based on the in vivo observation of the widened sutures/fontanelles, which is not dependent of chondrogenesis, and diminished AP expression in Col2Cre+/RosaIKK2ca homozygotes, it is possible that constitutive active IKK2 also suppresses osteoblast differentiation. To address this point, primary osteoblasts were isolated from new born IKK2ca transgenic and control counterparts and cultured them in osteogenic media. Similar to cultures of ST2 cells retrovirally infected with constitutive active IKK2, the kinase strongly suppresses primary osteoblast differentiation as shown by alkaline phosphatase staining of cells stimulated with osteogenic factor Wnt3a (fig. 3C). Real-time PCR also showed that IKK2ca downregulate Runx2 osteocalcin (OCN), osteoprotegerin (OPG) and alkaline phosphatase (AP) mRNA expression in IKK2ca-derived OBs (fig. 3D). In addition, IKK2ca expression led to increased expression of WNT antagonists; sclerostin (SOST) and DKK1, and increased expression of RANKL (fig. 3D). Both ex vivo and in vitro data demonstrate that constitutive active IKKβ suppresses osteoblast differentiation and skew RANKL/OPG ratio toward enhanced osteoclastogenesis.

### Expression of IKK2ca Impedes Bone Formation and Reduces Bone Mineral Density

**Figure 4:**
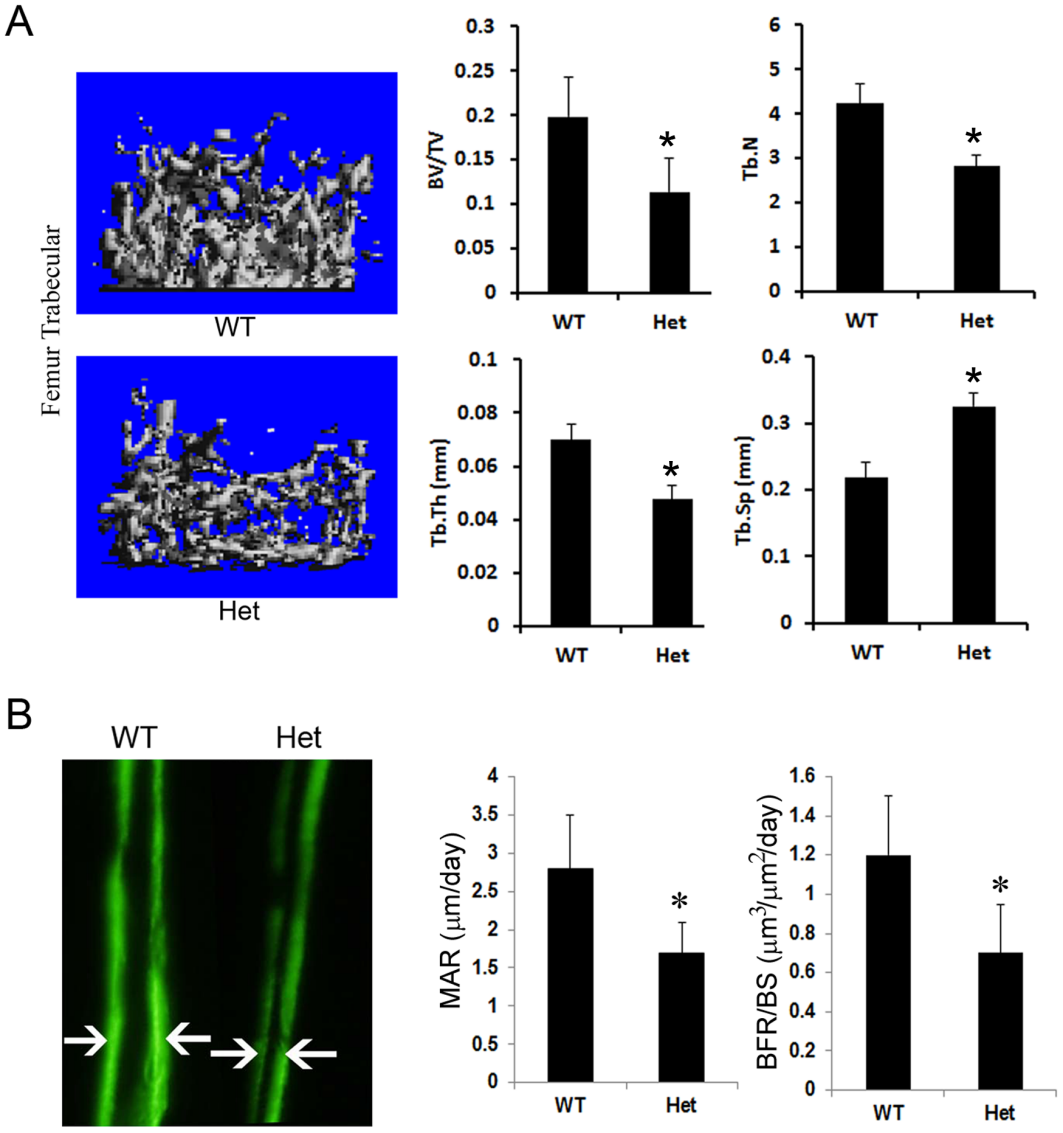
Expression of IKK2ca impedes bone formation and reduces bone mineral density. (A) Micro-CT analysis of four-week old Col2Cre+/IKK2ca heterozygote (Het) and WT littermate (n = 6/group). Col2Cre+/IKK2ca heterozygote has reduced trabecular bone in proximal tibia and femur compared to WT littermate. Quantitation of bone volume (BV) over total volume (TV), trabecular number (Tb.N), trabecular thickness (Tb.Th.) and trabecular separation (Tb.Sp.) is shown. (B) Bone formation rate - 4-week old mice (n = 6/group) were injected with two consecutive labels (arrows) of calcein (7 days apart) to measure bone formation rate. Asterisk represents p<0.05.

Inhibition of osteoblast differentiation markers by IKK2ca suggests that endogenous expression of the kinase in vivo may negatively impact normal skeletal development. To examine this possibility, we utilized IKK2ca heterozygous mice, which lived normally but exhibited moderate ossification defects (fig. 2) at developmental stages. Micro-CT analysis shows that mature heterozygous mice exhibited significant low bone mass (BV/TV), consistent with decreased trabecular thickness (Tb.Th) and number (Tb.N) and increased trabecular space (Tb.Sp) (fig. 4). To further establish this phenotype, dynamic bone markers such as bone formation rate and mineral apposition rate were measured using bone double labeling approach. The images depicted in figure 4B show that IKK2ca attenuated bone formation and exerts its inhibitory effect in an osteoblast-intrinsic fashion. We further provide preliminary evidence that osteoclast number is elevated in IKK2ca-expressing mice (Fig. S2), suggesting that bone resorption by osteoclasts may contribute to this phenotype.

## Discussion

Inflammation of bone negatively impacts its integrity and strength and alters bone-dependent metabolic pathways [21], [43]–[47]. Therefore, understanding the underlying mechanisms of this phenomenon in bone cells is critical for establishing countermeasures. The fact that NF-κB is central mediator of inflammatory responses positions this family of proteins and kinases as an attractive target for exploration. NF-κB proteins regulate numerous physiological and pathological processes, including the innate- and adaptive-immune responses, cell death and inflammation. The classical NF-κB pathway is driven by the activation of IKK2, which stimulates anti-apoptotic, pro-inflammatory and proliferative pathways, although pro-apoptotic functions have also been described [23], [24]. Furthermore, it has been reported that IKK2 functions through NF-κB-dependent and independent pathways. The choice of IKK2ca in our model to mimic sustained inflammation is based on the fact that activation of this kinase has been described as the converging point for all inducers of the canonical NF-κB pathway, which is the principal pathway that governs inflammatory processes.

**Figure 5:**
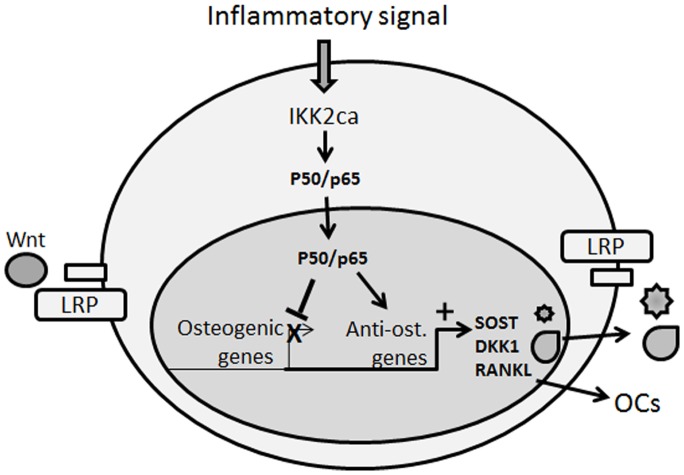
Proposed schematic model. IKK2ca mimics persistent NF-κB activation by inflammatory signals. In this model, however, activation of the NF-κB is restricted to the mesenchymal compartment using Coll2-cre driven expression of the active IKK2 form. IKK2ca leads to exacerbated expression of NF-κB subunits (indicated here as p50/p65). According to our data, NF-κB activation inhibits expression of osteogenic markers while stimulating expression of anti-osteogenic (SOST/sclerostin, DKK1) that antagonize Wnt binding to LRP and increasing pro-osteoclastogenic factors (RANKL) that stimulate osteoclast formation. The net effect of these actions is inhibition of bone formation and increased bone resorption.

In recent studies, we and others have examined the effect of NF-κB in the myeloid compartment. We demonstrated that constitutive activation of NF-κB led to significant bone loss in mice [31]. In the current study, we turned to examine the effect of active NF-κB in the mesenchymal compartment. Our results indicate that constitutive activation of NF-κB pathway using collagen-II cre knock-in in osteoblasts and chondrocytes perturbed osteogenesis and chondrogenesis. Considering both facets of NF-κB activation in myeloid and mesenchymal compartment, this approach nearly mimics systemic inflammation such as in rheumatoid arthritis wherein compromised osteoblast activity and exacerbated osteoclastogenesis is prevalent at inflammatory sites. Consistent with this notion, it has been reported that expression levels of several WNT antagonists are elevated in arthritic synovial fluid further contributing to inhibition of bone formation[17], [48]–[50]. Our data establish attenuation of osteogenic differentiation markers consonant with elevated levels of DKK1 and sclerostin (modeled in Fig. 5). We speculate that NF-κB-induced genes activate *sost* and *dkk1* genes leading to higher expression of their products, sclerostin and DKK1, respectively, which inhibit Wnt signaling in osteoblasts (Fig. 5). Ongoing studies in our laboratory are now focused on investigating the molecular mechanisms underpinning NF-κB-mediated inhibition of WNT signaling. In this regard, strong supporting evidence was recently described by Chang et al., [51] suggesting that NF-κB inhibits osteogenesis by promoting β-catenin degradation. This study, however, utilized the pleiotropic cytokine TNF to activate NF-κB and as a result cannot account for NF-κB-independent effects of the cytokine on osteogenesis.

Although activation of NF-κB is normally transient, chronic or sustained activation of NF-κB as is the case of our model described in this study leads to the establishment of inflammatory conditions through expression of a wide range of mediators including cytokines, radical oxygen species, and cyclo-oxigenases. This mode of NF-κB activation is associated with aging and has been described as a molecular culprit of inflamm-aging [52]. In this regard, secondary osteoporosis has been described as a co-morbidity of numerous low grade systemic inflammatory responses attending aging individuals. More importantly, a credible link between efficiency of NF-κB signaling and the level of inflammation has been established and is regulated by SIRT1 and FoxO, well-established regulators of aging [53], [54]. In fact, it is plausible that Sirt1 augments bone formation by down regulating NF-κB -induced SOST expression. Future studies will be required to confirm the molecular details of this proposition using experimental models of inflammatory-aging bone loss.

The increase in sclerostin and DKK1 expression levels and coincidental increase in RANKL/OPG ratio in IKK2ca-expressing mice (Fig. 5) is also reminiscent of similar expression profiles of these factors during bone fractures, suggesting that NF-κB plays a central role during bone injury and repair. Accordingly, during the initial phase of bone trauma/fracture a robust local inflammatory response, highlighted by enhanced levels of NF-κB activity [55], develops and encompasses recruitment of immune cells including macrophages to the injury site. At the conclusion of this stage, high levels of NF-κB are expected to subside and a bone reparative phase and angiogenesis govern the site. In support of this notion, it has been suggested that bone formation is often repressed in patients suffering from rheumatoid arthritis and that DKK1 and sclerostin (inhibitors of bone formation) were detected in serum of these patients and other inflammatory conditions [18], [19], [50], [56], [57]. Similarly, bacterially contaminated bone fractures fail to heal [58], suggesting that bacteria-derived lipopolysaccharides activate NF-κB in various cells, including osteoclasts and osteoblasts, and impede repair. Therefore, precise control of the duration and strength of NF-κB activity are crucial for modulating and resolving inflammatory osteolysis.

## Supporting Information

Figure S1 **IKK2ca inhibits mRNA expression of ST2 and ATDC5 differentiation markers.** ST2 and ATDC5 cells were infected with plasmids as indicated. Relative expression of alkaline phosphatase (from ST2 cell RNA), Sox9, Col2A1, and aggrecan mRNA (from ATDC5 cells) was measured at the time points shown.
(TIF)

Figure S2 **Histologic analysis of long bones.** Tibia from wild type or IKK2ca heterozygote (Het) were processed for histology and sections were immunostained with H&E (not shown) or tartrate-resistant acid phosphatase (TRAP) to detect osteoclasts. Number of osteoclasts per bone surface area (N.OCs/BS) from WT and Het sections is depicted.
(TIF)
