# Supplementary figures and images for: Constitutive Activation of IKK2/NF-κB Impairs Osteogenesis and Skeletal Development
Source: PLoS One. 2014 Mar 11;9(3):e91421. doi: 10.1371/journal.pone.0091421 (PMC3949987; doi:10.1371/journal.pone.0091421)

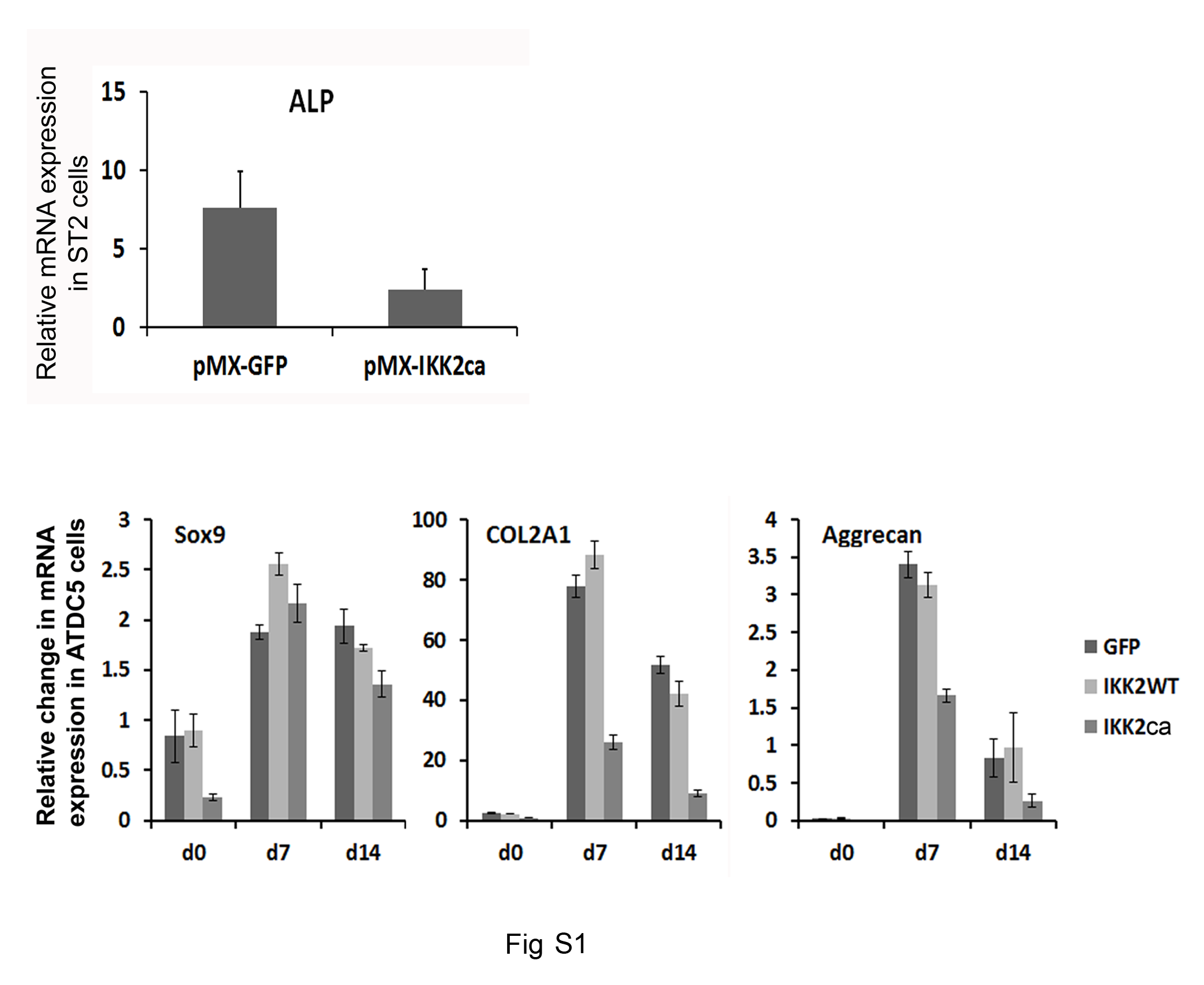

Supplement: Figure S1 — IKK2ca inhibits mRNA expression of ST2 and ATDC5 differentiation markers. ST2 and ATDC5 cells were infected with plasmids as indicated. Relative expression of alkaline phosphatase (from ST2 cell RNA), Sox9, Col2A1, and aggrecan mRNA (from ATDC5 cells) was measured at the time points shown. (TIF) [file pone.0091421.s001.tif]

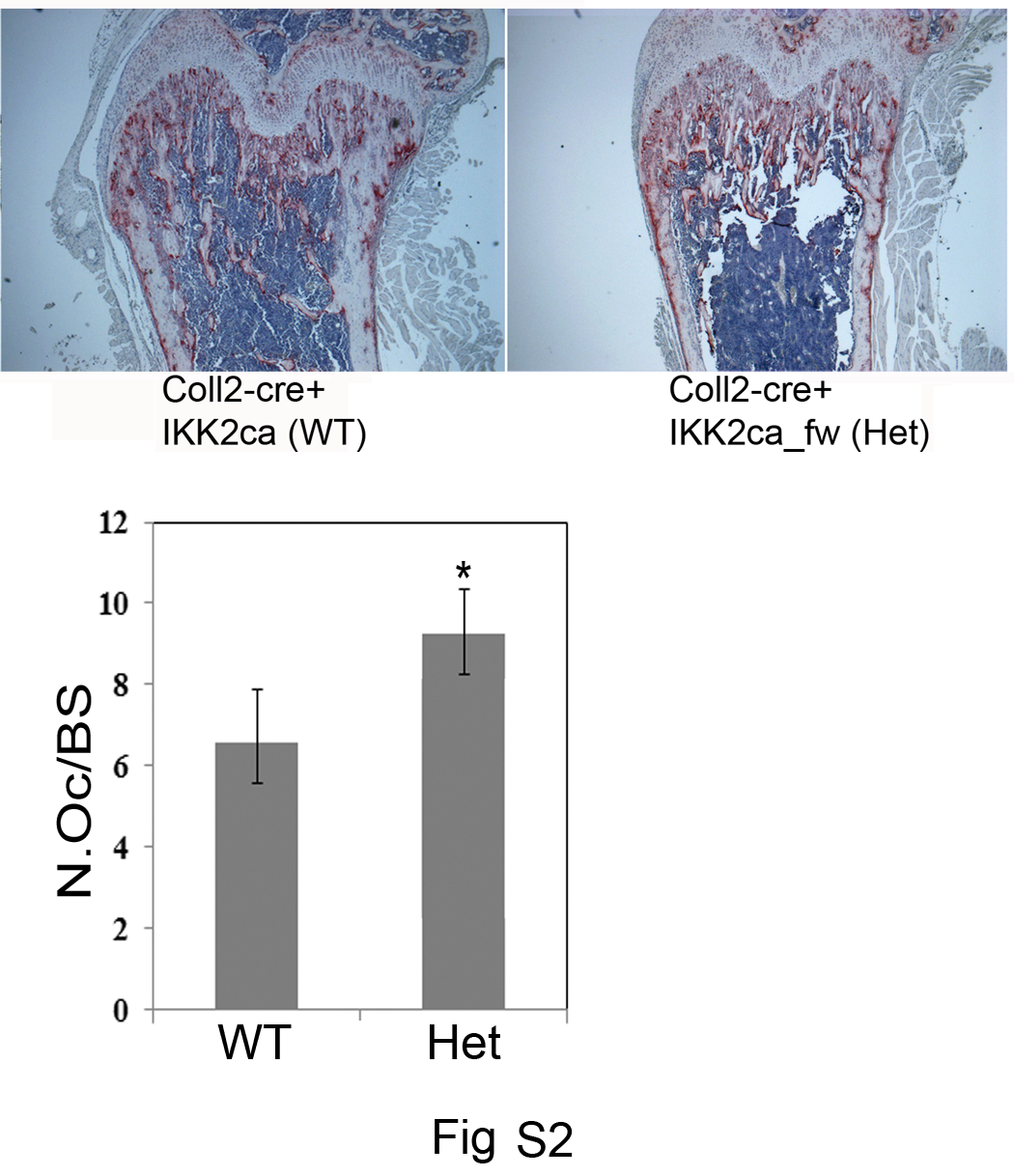

Supplement: Figure S2 — Histologic analysis of long bones. Tibia from wild type or IKK2ca heterozygote (Het) were processed for histology and sections were immunostained with H&E (not shown) or tartrate-resistant acid phosphatase (TRAP) to detect osteoclasts. Number of osteoclasts per bone surface area (N.OCs/BS) from WT and Het sections is depicted. (TIF) [file pone.0091421.s002.tif]
